# Global sharing of COVID‐19 therapies during a “New Normal”

**DOI:** 10.1111/bioe.13028

**Published:** 2022-04-25

**Authors:** Nancy S. Jecker, Caesar A. Atuire

**Affiliations:** ^1^ Department of Bioethics and Humanities, School of Medicine University of Washington Seattle Washington United States; ^2^ Department of Philosophy University of Johannesburg Johannesburg Gauteng South Africa; ^3^ Centre for Bioethics Chinese University of Hong Kong Shatin New Territories Hong Kong; ^4^ Department of Philosophy and Classics University of Ghana Legon Accra Ghana; ^5^ All Souls College University of Oxford Oxford United Kingdom of Britain and Northern Ireland

**Keywords:** allocation, COVID‐19, ethics, global health, justice, solidarity

## Abstract

This paper argues for global sharing of COVID‐19 treatments during the COVID‐19 pandemic and beyond based on principles of global solidarity. It starts by distinguishing two types of COVID‐19 treatments and models sharing strategies for each in small‐group scenarios, contrasting groups that are solidaristic with those composed of self‐interest maximizers to show the appeal of solidaristic reasoning. It then extends the analysis, arguing that a similar logic should apply within and between nations. To further elaborate global solidarity, the paper distinguishes morally voluntary, sliding‐scale, and mandatory versions. It argues for an all‐hands‐on‐deck approach and gives examples to illustrate. The paper concludes that during the COVID‐19 crisis, global solidarity is a core value, and global sharing of COVID‐19 treatments should be considered a duty of justice, not of charity.

## 1 INTRODUCTION

The 2019 coronavirus disease (COVID‐19) pandemic is an evolving crisis that experts predict will eventually transition to an endemic state. If this occurs, excess deaths attributed to the novel coronavirus will decline over time, and the emphasis will gradually shift to include not just preventive strategies such as vaccinating, testing, tracing and masking, but also the ongoing management of people who become infected. While preventive strategies remain a crucial component of national responses to COVID‐19, it has become increasingly clear that more than prevention is needed to cope effectively and manage life with COVID‐19 long‐term. What some have termed the “new normal” has begun to take shape.^1^ In the new normal, the battle to eliminate COVID‐19 is given up, and the new aim is learning to live alongside the novel coronavirus, in much the same way that we have learned to live alongside other circulating respiratory viruses often referred to as “the common cold.”^2^ Some see the new phase as putting an end to “COVID‐19 exceptionalism,” which focuses narrowly on a single disease while sidelining the full range of threats to human health.^3^ Others describe it as a change “from crisis to control.”^4^ Still others say that a new phase commences after the pandemic threat is subdued and “total respiratory viral infections, hospitalizations, and deaths inclusive of those from COVID‐19 are no higher than what typically occurred in the most severe influenza years before the current pandemic.”^5^ Yet, even while we remain in the pandemic phase, we are already witnessing a greater emphasis on treating patients with COVID‐19 as new options become available.

One reason for underscoring treatment as well as prevention is that even as global access to vaccines expands, many people refuse vaccines and enter the hospital with severe disease. In an ongoing global survey, Johns Hopkins University researchers reported that more than half of unvaccinated people in more than 50 countries say they definitely or probably would not get a COVID‐19 vaccine.^6^ In addition, the risk of COVID‐19 after vaccination—so called “breakthrough cases”—has become increasingly common as new and more transmissible variants of concern have emerged.^7^ Omicron is a case in point. Designated a variant of concern on November 26, 2021, most currently available vaccines provide only limited protection against the variant.^8^ In addition, Omicron may be less reliably detected with commonly used COVID‐19 diagnostic tests, resulting in people testing negative and unwittingly infecting others.^9^

When people become infected with the SARS‐CoV‐2 virus, the care available to them depends on many factors, including not only the supply of therapeutics, but also the supply of healthcare providers, personal protective equipment, and hospital beds, as well as transportation to access healthcare services and insurance coverage or the ability to pay for care. We focus on the supply of COVID‐19 treatments, specifically treatments we call *basic medicines*. These include COVID‐19 treatments for which there is at least preliminary evidence showing significant reduction in severe disease and death among infected people and an ability to administer treatment on an outpatient basis or as part of a short‐term hospital stay. These kinds of treatments are most amenable to sharing globally because they can more readily be used in under‐resourced settings, such as low‐ and middle‐income countries (LMICs) with little or no intensive care unit capacity and limited capacity for inpatient hospital care.

The question of global sharing arises because basic COVID‐19 medicines are subject to both chronic and episodic shortages. Chronic shortages can develop in some parts of the world as the result of intellectual property protections that give pharmaceutical companies the ability to control production and set prices that poorer countries can barely afford, thereby limiting supply.^10^ The same protections enable drug companies to limit global supply by not sharing recipes and licenses for manufacturing COVID‐19 treatments. Episodic shortages can occur when new variants of concern render some, but not all, treatments ineffective. For example, with the Omicron coronavirus, preliminary studies showed that two of three authorized monoclonal antibodies for treatment and post‐exposure prophylaxis, Regeneron (casirivimab/imdevimab) and Lilly (bamlanivimab/etesevimab), lacked efficacy.^11^ Only GlaxoSmithKline's Sotrovimab retained activity.^12^ As of January 7, 2022, worldwide supply of Sotrovimab was extremely limited.

With few exceptions,^13^ global sharing of basic COVID‐19 treatments has not received the attention it deserves. Most bioethical debate about the global allocation of COVID‐19 resources has addressed vaccines,^14^ rather than treatments. This paper fills this gap and asks, ‘Do countries have a duty to share basic COVID‐19 treatments post‐pandemic?’ Even after the pandemic phase of COVID‐19 ends, new variants of concern may emerge, and a global crisis may ensue. If crises occur, what ethical values ought to govern? If there is a duty to share COVID‐19 treatments, what values give rise to this duty? Is it a strict requirement of justice or a voluntary benevolence‐based duty? How can it be operationalized? We address these and related questions in a stepwise fashion. Section 2 distinguishes two types of COVID‐19 treatments and models sharing strategies for each in small‐group scenarios, contrasting groups that are solidaristic with those composed of self‐interest maximizers to show the appeal of solidaristic reasoning. Section 3 extends the analysis, arguing that a similar logic applies within and between nations. Section 4 further elaborates global solidarity by distinguishing morally voluntary, sliding‐scale, and mandatory versions. The paper concludes (in Section 5) that an all‐hands‐on‐deck approach is required to operationalize solidarity during the COVID‐19 pandemic phase and beyond, giving examples to illustrate. Our focus throughout the paper is not to provide a comprehensive framework for global allocation for COVID‐19 treatments, but rather to identify solidarity as a core value to be considered.

## 2 SMALL SOLIDARISTIC GROUPS

We divide basic COVID‐19 medicines into optimal and suboptimal types based on their ability to reduce severe disease or death.^15^ While we give specific illustrative examples, the science behind COVID‐19 treatments is in its infancy. New treatments will continue to emerge, sometimes replacing those we identify. Our examples thus serve only as placeholders and can be substituted with other therapeutics with similar benefit profiles. *Optimal therapy* refers to preferred treatments for patients that reduce severe disease and/or death to the greatest extent. These are the most beneficial treatments for a particular patient population. Today, optimal therapy includes Pfizer's Paxlovid and Merck's Molnupiravir, which can reduce the risk of severe disease or death in high‐risk patients. It also includes monoclonal antibody therapeutics and steroids. *Suboptimal therapy* refers to treatments that are less beneficial than the preferred treatments and that reduce severe disease and/or death to a lesser extent. These treatments do not include modalities that are currently considered optimal—for example, Remdesivir or monoclonals would not be offered to patients who would otherwise qualify. Instead, today's suboptimal therapy might include steroid and supplemental oxygen.

With these distinctions in mind, suppose you belong to a solidaristic group (Group 1). It could be a four‐person family consisting of healthy parents in their 50s and high‐school children aged 16 and 17. Each member of the group has become infected by a highly transmissible mutation of the SARS‐CoV‐2 virus. Since each person was previously healthy, their risk of severe disease and death is more‐or‐less equal. Suppose that preliminary clinical trial data show that 80% reduction in severe disease or death against a new coronavirus variant is provided by optimal therapy, and 60% by suboptimal treatment. Relying just on that simplified description, what would be the right thing to do if affording optimal therapy to everyone was not possible? More supply might be available later, but future allotments are uncertain. For simplicity, imagine that the group has limited resources and can invest them in one of two ways: optimal treatment for one person or suboptimal treatment for two people.

If Group 1 is inlined to be solidaristic, then when shortages like this arise, its members would offer to help one another. For example, parents might opt to give their two teenage children some protection, while foregoing protection for themselves. It might be thought that parents have a duty to sacrifice on their children's behalf, foregoing treatment for themselves unless their risk is significantly greater than their children's. If their children were younger and more dependent on their care, however, they may prefer to provide some protection to one child and one parent based on a consensus about who could best care for them. The alternative of giving one person optimal treatment and others nothing would not be considered an ethically viable option.

Yet, giving optimal protection to one member of a group and others no protection might be considered in a different solidaristic group, Group 2. Group 2 includes four people with COVID‐19 who have different risk levels, with one member having a much higher chance of severe disease or death. For example, if Group 2 was a multigenerational family with two parents in their 50s, a teenager, and a 65‐year‐old, they may opt to give the most protection (optimal treatment) to the 65‐year‐old, with the other three people going without (Strategy 1). Alternatively, they may choose to give some protection (suboptimal treatment) to the 65‐year‐old and one of the parents in their 50s. When deciding which middle‐aged adult to protect, Group 2 might seek consensus based on what was best for the group as a whole (Strategy 2). Failing consensus, they might use a random method, say flipping a coin. As shown in Table 1, the greater protection afforded the older adult would make Strategy 1 a better allocation for the group as a whole, as well as for the 65‐year‐old. The greater absolute risk to the child and middle‐aged adults of Strategy 2 compared with Strategy 1 (0.1% and 1.0% respectively, as shown in Table 1) is small; it does not place them at great peril, even though it increases their relative risk.

**TABLE 1:** Hypothetical risk for severe disease or death with different allocation strategies

		Strategy 1: Inpatient treatment for 1 person (older adult)	Strategy 2: Outpatient treatment for 2 people (1 middle‐aged adult and 1 older adult)	Strategy 1 vs. 2
	Pre‐treatment risk	Post‐treatment risk	Post‐treatment risk	Absolute risk reduction
**Child**	0.1%	0.1%	0.1%	0
**Middle‐aged adult 1**	1%	1%	1%	0
**Middle‐aged adult 2**	1%	1	0.4%	0.6%
**Older adult**	10%	2%	4%	−2%
**Group**	12.1%	4.1%	5.5%	1.4%

The proclivity to help others in solidaristic groups reveals an allocation strategy, *donation*, which involves helping others by reducing one person's protection under conditions of resource scarcity. In Group 1, donation was used when parents offered to protect their children and go without. In Group 2, donation was applied when the parents and child offered to go without to protect the older adult who faced relatively higher risk, and when one parent and one child offered to go without to protect a larger number of people (two people rather than one person).

Notice that a decision to donate to give more protection to others could be reached by appealing to utilitarian reasoning. However, a key difference between utility maximizers and solidaristic deliberators is that utility maximizers are unconcerned with how benefits get distributed; they focus just on maximizing aggregate good. For this reason, utility maximizers would help those facing serious threats to their health and life only if doing so maximized aggregate benefits. By contrast, solidaristic deliberators show concern not only for the collective “we” but for individuals as such, making reasonable efforts to distribute limited resources in ways that help those in greatest peril reach a minimal threshold of protection.

To further elaborate solidaristic reasoning, consider a scenario in which only suboptimal protection is available, and the supply is only enough for three people. Group 1 (where all members have roughly equal risk) might allot each of the two children protection and, for the remaining treatment, seek consensus; failing consensus, they might draw straws, reflecting an underlying belief that each person is valued equally. Group 2 (where risks are not equal) might divvy up their three treatments to give priority to those at greatest risk, which would be the older adult and two middle‐aged parents.

We might juxtapose these solidaristic groups with a different kind of group, Group 3, which is not solidaristic, but instead is composed of self‐interested maximizers. In Group 3, there exists little mutual concern among members. When COVID‐19 treatments are limited, conflicts are apt to arise about which eligible person should receive priority, with each vying to put their individual interests ahead of others. In this group, each member presses for the highest protection possible (optimal treatment), rejecting lesser protection, irrespective of their risk relative to others in the group.

## 3 SOLIDARITY WITHIN AND BETWEEN NATIONS

How do solidaristic strategies and their underlying ethical bases relate to the policies of nations? In small solidaristic groups, like families, people's lives are interconnected in the sense that what happens to one person profoundly impacts what happens to others. Bonds of love and caring unite members of the group, leading them to take each other's interests into account. Yet these same interconnections do not bind strangers who have never met.

Yet, despite such differences, people who have never met are nonetheless interconnected in other relevant respects, and solidaristic reasoning continues to apply. During an infectious disease outbreak like the COVID‐19 pandemic, all people share a vulnerability to infectious disease or death, even though their levels of risk may differ. Such interconnectivity is apparent everywhere people meet—in schools, shops, restaurants, gyms, and workplaces, because shared spaces create pathways for disease spread and the prospect of disease and death. Francis et al. propose that rather than thinking of people as residing safely inside impermeable bodies, during an infectious disease outbreak people are more aptly thought of as “victims and vectors, ill because of something that came from others and could go to others.”^16^

During an infectious disease outbreak, “solidarity” applies in the most basic sense of the word: individuals are “united or at one in some respect, especially in interests, sympathies, or aspiration.”^17^ Solidarity in this sense is not derived from essential features of humanity, but from concrete recognitions of similarities in a specific context.^18^ In the context of a pandemic, all share susceptibility to disease and death from a contagious pathogen. Prainsack and Buyx characterize solidarity as also including a response, which takes the form of “an enacted commitment to carry ‘costs’ (financial, emotional, or otherwise) to assist others with whom a person or persons recognize a similarity in a relevant respect.”^19^ A group of people enact solidarity during a pandemic when they are willing to expose themselves to risk to assist others with whom they share a common vulnerability.

Building on these ideas, we propose that a commitment to solidarity during the COVID‐19 pandemic emphasizes certain core values for nations that were revealed earlier, in the deliberations of Groups 1 and 2: (1) *equality*, or a duty to consider each person's interests equally and to avoid prioritizing one over another, other things being equal;^20^ (2) *the common good*, or a duty to protect the well‐being not just of each individual, but of the group as a whole;^21^ and (3) *special duties*, or duties to the least advantaged, especially when the situation of the least advantaged falls beneath a minimal threshold of protection.^22^ Regardless of how these values are specified and ordered, together they point, like a compass, to the direction we ought to take when allocating a limited supply of basic COVID‐19 therapies within and between nations. At the national level, solidaristic thinking lends credence to allocation policies that assign priority for limited treatments to infected people with the greatest risk of severe disease and death. This reasoning is in some respects similar to the criteria proposed in frameworks for domestic^23^ and global^24^ allocation of COVID‐19 vaccines that identify high risk of infection and/or high risk of severe disease and death as allocation criteria.

Rather than taking the stance that each person should seek to optimize their own situation, shown by the self‐interested maximizers in Group 3, protecting the whole (based on concern for the common good) becomes an ethically overriding consideration. So too does lending a hand to the most disadvantaged people (based on special duties), who experience a higher risk of severe disease and death, whether due to age, health condition, or social conditions associated with systemic racism or poverty.

The same logic that justifies sharing COVID‐19 therapies within nations gives warrant to sharing between nations and striving to protect all people against the potentially debilitating and deadly effects of the SARS‐CoV‐2 virus. We might think of nations as being like members of a solidaristic group. Their solidarity arises from globalization, or the “movement of people, goods, services, and ideas across a widening set of countries,” which impacts not just the goods and services people consume, but also the microbial world they inhabit.^25^ As people move across borders, they create a shared microbial space, or what has been called “a natural world, sans borders.”^26^ In a globally interconnected space, a threat to one member poses systemic threats to the whole interconnected group. The spread of the Delta variant of concern serves to illustrate. After the initial detection of Delta in India, it rapidly spread across the world, creating resurgences of COVID‐19 wherever it landed, and spreading at a rate consistent with its being roughly 60% more transmissible than the already highly infectious Alpha variant.^27^ In the context of the Delta variant, dichotomies between “us” and “them” distort our thinking, glossing over the fact that what happens in one place potentially affects the health and life of people everywhere. Just as poltants in the sky and degradation of the Earth endanger people everywhere, the SARS‐CoV‐2 virus anywhere threatens people everywhere. *In these kinds of situations, the ethic that ought to govern nations is solidaristic*.

Admittedly, the challenge of protecting all the nations of the world is daunting. There are 7.9 billion people. Solidaristic reasoning suggests that in a situation of limited resources, priority should in principle go to those with the greatest risk wherever they reside. This conclusion gains support from all three solidaristic principles: *equality*, *common good*, and *special duties*. To illustrate, consider a simple hypothetical exmple, shown in Table 2. In this example, we suppose that wealthy nations have sufficient supplies of the best available COVID‐19 treatment to protect all their citizens many times over, while LMICs have a limited supply of less beneficial treatments for people at high risk; they have no treatment for people with other risk profiles.

**TABLE 2:** Solidarity and self‐interested maximizing strategies

		(1) Self‐interested maximizing: priority to one's o wn citizens	(2) Solidarity: priority to people at greatest risk wherever they are	(1) and (2) compared
**Pre‐treatment risk of severe disease/death, 1000 individuals**		**Post‐treatment risk of severe disease/deathp**		**Disease/death reduction**
**Children 0.1%**	LIC	1 (no treatment)	1 (no treatment)	0
	HIC	.33 (treatment)	1 (no treatment)	−0.67
**Older adults 10%**	LIC	100 (no treatment)	10 (treatment)	90
	HIC	10 (treatment)	10 (treatment)	0

*Note*: HIC, high‐income country; LIC, low‐income country. Assumes one‐third reduction in disease/death for treated children and one‐tenth reduction in treated older adults.

If wealthy nations act like self‐interested maximizers, they will hoard their supply for their own population. For example, an allotment of 1000 doses of the best available treatment might be given to previously healthy children in high‐income nations who become infected with the SARS‐CoV‐2 virus, despite their low risk of becoming severely ill or dying. Meanwhile, high‐risk groups such as older adults in LMICs would go without optimal protection. Self‐interested maximizing would provide only an incremental benefit to wealthy nations, preventing severe disease and death in a relatively small number of people (20 children total, based on 0.02% × 1000 people). If wealthy nations instead opt for solidarity, this provides far more benefit. For example, if the 1000 doses of optimal treatment went to older adults, this would prevent 40 times more severe disease and death, helping 800 older people (8% protection × 1000 people) rather than 20 younger people. The broader lesson is that solidaristic thinking saves more lives and reduces more severe disease and death.

Is there any ethical justification for wealthy nations to hoard SARS‐CoV‐2 treatments for their own citizens? One line of thinking holds that children's lives have greater value because they have on average more future life years ahead to live. However, even taking this into account, the large difference in risk profiles between age groups means that more life years are saved by protecting older adults. For simplicity, suppose the children are healthy 15‐years‐olds, and the older people are healthy 65‐year‐olds, and suppose that each person will live for 75 years in total. Saving the 20 teenagers would result in saving 1200 life years (60 years saved × 20 people), while saving the 800 older adults would result in saving 8000 life years (10 years saved × 800 people).

Another ethical justification for wealthy nations holding on to their supplies and distributing them to their citizens with relatively lower risk emphasizes morbidity, rather than mortality, and claims that for some COVID‐19 treatments, such as treatments managing symptoms of long‐COVID, priority should go to younger people, because they would suffer for many more years on average. In reply, we leave open the possibility that duration of benefit, as well as severity of disease and risk of death, might be ethically relevant to priority setting. However, decisions about age‐related priorities should occur at other levels of allocation, since there are different cultural views about the relative priority that should be accorded to younger and older people.^28^

It might also be argued that wealthy nations have stronger special duties to protect their own people by keeping, or even stockpiling, COVID‐19 treatment for their own citizens. Schaefer et al. reason along these lines when they introduce an *influenza standard* for sharing COVID‐19 vaccines, which might be adapted and applied to COVID‐19 therapeutics. It would then say that when COVID‐19 becomes more like a bad influenza season in terms of mortality, other health effects and public health restrictions, then there is no longer an ethical justification for retaining COVID‐19 therapeutics for residents of one's own country.^29^ Yet our analysis supports an adapted version of what Jecker and Lederman dub a *fair‐minded influenza standard*: until COVID‐19 resembles a bad influenza season, governments should work cooperatively, sharing COVID‐19 therapeutics.^30^

Schaubroeck and Hens cast doubt on solidaristic reasoning for another reason. They claim that differences have become more glaringly apparent than similarities during the pandemic—between rich and poor, Black and White, the global North and the global South.^31^ Perhaps, global solidarity sets the bar too high. Could it ever be realized? In response, a shift to more global and solidaristic thinking is a process that unfolds over time. It begins with a dawning awareness of an issue; proceeds toward better understanding by working through the issue, sometimes in fits and starts; and finally leads to resolution on cognitive, emotional and moral levels.^32^ The pandemic has raised awareness of global health disparities; we are now in the midst of understanding and working through them. The highly transmissible Omicron variant serves to illustrate. After its initial detection in South Africa and Botswana, some countries reacted by banning all travelers from southern Africa.^33^ However, there is little evidence that travel restrictions reduce disease spread, since they are seldom timed right and they are paired with other preventive strategies.^34^ It soon became apparent that Omicron was already circulating widely outside southern Africa, revealing the flaw of “us–them” logic during an infectious disease outbreak. The WHO's Regional Director for Africa, Matshidiso Moeti, reframed the travel bans in the language of solidarity, which made better sense of the situation. “Travel bans that target Africa attack global solidarity,” Moeti said, pointing out that “COVID‐19 constantly exploits our divisions. We will only get the better of the virus if we work together for solutions.”^35^ Others who invoked solidarity regarded Omicron as a clarion call to vaccinate the world and minimize future threats. The WHO Director‐General, Tedros Ghebreyesus, for example, responded to Omicron by urging the world to prioritize helping all countries to vaccinate 40% of their populations as quicky as possible and 70% by the middle of 2022, in order to minimize the chance of future variants of concern emerging;^36^ solidaristic thinking underpinned the Director‐General's approach.^37^ Bioethics can contribute to galvanizing people to show solidarity by distinctly articulating it as a core value and applying it in concrete ways that people understand.

Finally, it might be argued that *enlightened* self‐interest maximizers in wealthy nations would reach the same conclusion as solidaristic reasoners did in our example (shown in Table 2). They would consider their *long‐term* self‐interest and wish to avoid dangerous mutations that could arise by not treating high‐risk people.^38^ In reply, political winds often steer self‐interested maximizers to focus on shorter‐term goals. This is because political leaders who reason in accordance with self‐interested maximizing apply this reasoning to their own case. In democratic states, this translates into leaders maximizing their odds of re‐election, while in non‐democratic states, it might mean burnishing one's image to tighten one's grip over a nation. By contrast, solidarity by its very nature is not about me or my political aspirations. It steers a course directed at helping a larger group of people. In small groups, like families, parents think not only about themselves or even their children, but about their grandchildren and future generations of their family. National governments and international leaders that embrace solidaristic values acquire a longer‐range, wider‐angle view. Solidarity at this level invites thinking about people who are geographically and temporally distant. When nations act in a solidaristic fashion within their borders, they afford a social safety net that ensures that people can access a minimal threshold of protection, independent of their ability to pay and based on their level of risk.^39^ When international leaders deliberate in a solidaristic way, they make reasonable efforts to ensure that people everywhere have a minimal threshold of protection, regardless of their country of origin. They recognize special duties to those at greatest risk—in this case, people in poorer nations at high risk of severe disease and death from the SARS‐CoV‐2 virus.

## 4 TRANSLATING GLOBAL SOLIDARITY

Solidaristic deliberation applied to global sharing of COVID‐19 therapies can be operationalized in a range of ways.

### 4.1 Voluntary solidarity

One option is what Gunson calls “weak solidarity,”^40^ and which we call “voluntary solidarity.” Voluntary solidarity holds that showing solidarity is a voluntary duty, like charity.

According to voluntary solidarity, once basic COVID‐19 treatment for people at high risk is reasonably available within a nation, that nation has a duty of charity to share basic COVID‐19 treatments with other nations who lack this level of protection. Charitable contributions could be coordinated by an international organization overseeing procurement and distribution.

Voluntary solidarity was evident, for example, in Merck's agreement with Medicines Patent Pool to share a royalty‐free license for its COVID pill, Molnupiravir, with 105 low‐income countries, mostly in Africa and Asia.^41^ However, a limitation of voluntary solidarity is that it sets a low bar because it allows nations, drug companies, civil society groups, and others to put off charity for another day. It does not hold people to any specific standard. Charity‐like approaches can also spawn dependence among recipients, weakening, rather than strengthening, their capacities for future agency. By contrast, solidarity encourages people to stand together and be mutually responsible for helping one another.

### 4.2 Sliding‐scale solidarity

A second possibility identifies duties toward compatriots and people beyond borders and seeks to balance them, usually in a way that leans heavily toward compatriots. This view is suggested by moderate nationalists, such as Scheffler, who recognize duties to people everywhere, yet consider duties to fellow citizens stronger.^42^

According to sliding‐scale solidarity, once basic COVID‐19 treatment for people at high risk is reached within a nation, that nation has a duty of charity to donate a percentage of its supply of basic COVID‐19 treatments to other nations with high‐risk groups that lack such protection. This could be coordinated by an international organization overseeing procurement and distribution. Additional supplies can be used for compatriots with lesser needs.

Like voluntary solidarity, sliding‐scale solidarity is based on principles of humanitarian benevolence and compassion, but it goes further than voluntary charity by setting a specific target, often a percentage of total product, and commits to it. The target set is a sliding scale, which asks more of countries with relatively greater wealth and less of countries that have less. For example, the U.S. National Academies of Science, Engineering and Medicine took this approach to the domestic allocation of globally scarce COVID‐19 vaccines, recommending deploying a portion (e.g., 10%) of U.S. vaccine supply for global allocation, “both as a means to help contain the pandemic and as an effort to build global solidarity in addressing this pandemic—and the next.”^43^ Sliding scale solidarity was also evident in 2009, when the U.S. government committed to give 10% of its H1N1 influenza vaccine to the WHO to promote global development and create an international climate conducive to cooperation and the sharing of viral samples and genetic sequences. Yet accountability remains a problem for sliding‐scale solidarity because it is still unenforceable, a duty of charity, not justice.

### 4.3 Solidarity as justice

A third possibility envisions solidarity as a matter of justice. Gould suggests this view when describing solidarity as a “requirement to realize justice through solidaristic activity [which] arises from people's interdependence and the fact that their free development as agents requires a set of conditions, both material and social.”^44^ Jecker suggests this conception of solidarity when characterizing global health disparities during the COVID‐19 pandemic as arising in part from historical injustices in the global basic structure.^45^ Solidarity as justice also aligns with discussions of social capital that regard trust and social cohesion as integral to managing crises and propose integrating these values into public health policies.^46^

According to solidarity as justice, once basic COVID‐19 treatment for people at high risk is reached within a nation, that nation has a duty to share its excess supplies with other nations who are less well protected until access to a certain threshold level of protection is available in all nations.

When global sharing of COVID‐19 therapies is seen as a duty of justice, it is morally mandatory, enforceable, and applies impartially.^47^ Unlike charity, solidarity as justice springs from recognition of similarities, rather than of differences.^48^ During the COVID‐19 pandemic, solidarity surfaces from people's common stake in avoiding illness or death from the SARS‐CoV‐2 virus. By contrast, charity arises from a perception of difference—the haves perceive others as different, that is, as more needy and vulnerable. The approach of solidarity as justice was apparent pre‐pandemic, when a Lancet Commission recommended utilizing international law to promote global health justice, calling strong legal capacity “a key determinant of progress towards global health and sustainable development.”^49^ Solidarity as justice is also evident in efforts to use the treaty powers of the WHO to address global vaccine equity through an international pandemic treaty, first proposed by the President of the European Council, Charles Michel. According to Michel, such a treaty should be anchored in the value of solidarity, with the objective “to do better in all areas where we recognize it is in our interest to strengthen cooperation.”^50^

Part of the argument for solidarity as justice comes from sober recognition that the world has changed, and the “old” bioethics no longer suits the situation we are in. As ten Have argues, “the global dimension of bioethical issues has produced … a new kind of bioethics … It has a broader agenda and theoretical framework than bioethics as it has developed in the last half century.”^51^ In contrast to the bioethics of the past, which developed in the aftermath of the WWII as a response to revelations of egregious violations of individual rights, the global bioethics in the 21st century is a response to worldwide threats to health, such as the rise of emerging infectious diseases and zoonoses, climate change, and antimicrobial resistance. These issues foreground people's relationality, their ability to harm and be harmed by others. Addressing them requires expanding the repertoire of bioethical values in ways that better incorporate the common good and public interest, which solidarity as justice does.

Notably, all three accounts of solidarity are compatible with limited national partiality. Both voluntary and sliding‐scale solidarity prioritize compatriots at high risk of severe disease and death over others at high risk, but they do so while simultaneously recognizing cross‐border responsibilities. Solidarity as justice prioritizes high‐risk people within one's own country over high‐risk people elsewhere, but foregoes prioritizing people at lesser risk until a threshold level of protection of people at high risk in other countries is available.

It might be objected that when COVID‐19 treatments are globally shared, sending nations have little control over how receiving nations allocate them. What if receiving nations do not prioritize people at high risk of severe disease and death within their borders? In response, our arguments emphasize solidarity at the global level, specifying it in terms of ensuring sufficient protection to every nation; it is consistent with our argument that “sufficient protection” may mean different things at national and local levels, and may be differently specified in those settings. For example, some countries may choose to prioritize COVID‐19 treatments for groups who are at high risk of infection and provide vital services during an emergency, such as healthcare workers, even when those individuals have less risk of severe disease and death than others do.^52^

It might be argued that our analysis glosses over a larger philosophical debate between cosmopolitanism and nationalism,^53^ making quick work of a complex array of issues. Yet this response is mistaken. Our analysis suggests that it is misleading to characterize global sharing of COVID‐19 treatments as a choice between nationalism and cosmopolitanism. Instead, during a pandemic emergency, nationalism and cosmopolitanism *converge*. Ending the pandemic sooner benefits everyone, and this requires generating the global goodwill required to encourage acts of cooperation, for example sharing viral samples and genomic sequences when new variants of concern emerge. Sharing COVID‐19 treatments with people at high risk of severe disease and death is a duty of justice that lasts as long as SARS‐CoV‐2 remains a global threat to people everywhere. Even after the crisis phase becomes less severe, solidaristic arguments continue to apply, and sharing COVID‐19 treatments should continue as a crucial way to strengthen global cooperation and face down future pandemics and ongoing global health threats. An opponent of solidarity as justice might argue that unlike sharing vaccines, which benefits many people, sharing treatments helps only the individual receiving treatment. For this reason, the logic of “us” versus “them” is more suitable for treatments than solidarity. Yet, as suggested, sharing COVID‐19 treatments benefits everyone by creating the global goodwill crucial to confronting not only the COVID‐19 pandemic but other 21st century global health threats. Global sharing of COVID‐19 therapies also helps others by reducing the impact of high rates of severe disease and death in a population, which can disrupt global supply chains, wreaking havoc on global economies and people's livelihoods. Finally, when protections are not shared with people at high risk of severe disease and death, these people can go on to suffer prolonged bouts of disease, raising the risk of multi‐mutational SARS‐CoV‐2 variants arising over the course of their disease that can be shared with others through viral shedding late in a person's disease course; these mutations may be more contagious, virulent, or resistant to protections afforded by vaccines and treatments.^54^

To implement global solidarity, we endorse an all‐hands‐on‐deck approach. Not only the leaders of nations but also the leaders of international philanthropic organizations, multinational pharmaceutical companies, civil society groups, and others should commit to the global sharing of COVID‐19 therapies and translate this commitment into action. High‐income countries can promote solidarity by leveraging their purchasing power with pharmaceutical companies to make bilateral deals conditional on drug companies making treatments available at an affordable price (or for free) to poorer countries. For‐profit pharmaceutical companies can exhibit solidarity by committing to make bilateral deals transparent and publicly accountable and by capping the share of products available for bilateral purchase based on a country's needs.^55^ Over the longer haul, they can also share knowhow, transfer technology, and help build capacity in LMICs. International philanthropic organizations and civil society groups can demonstrate solidarity by helping to distribute treatments to people on the ground and investing in capacity building. Fully realizing solidarity at any level—voluntary, coordinated, or mandatory—will require more cooperation than the world has so far witnessed.

## 5 CONCLUSION

In conclusion, during the COVID‐19 pandemic, people who become infected with the novel coronavirus require treatment to reduce their risk of severe disease and death. As new treatments become available, both chronic and episodic global shortages of treatments may occur, raising questions about their just allocation. We have argued that solidarity ought to guide the allocation of globally scarce COVID‐19 treatments. During a pandemic and beyond, showing solidarity is a duty of justice, not charity. Emphasizing solidarity during a crisis builds social capital necessary for long‐term recovery after the crisis subsides. The “new normal” for COVID‐19 must reflect not just a change “from crisis to control,” but a reorientation of *ethical stance and attitude*: from “me first” to “us together.”

## Footnotes

1 Emanuel, E. J., Osterholm, M., & Gounder, C. R. (2022). A national strategy for the “New Normal” of life with COVID‐19. *Journal of the American Medical Association*, *327*, 211–212. Epub ahead of print, January 6: https://doi.org/10.1001/jama.2021.24282

2 Jecker, N. S., & Au, D. K. S. (2021). Does zero‐COVID neglect health disparities. *Journal of Medical Ethics*, *48*, 169–172. Epub ahead of print: https://doi.org/10.1136/medethics-2021-107763

3 Galea, S. (2021). Reckoning with COVID‐19 exceptionalism. *JAMA Health Forum*, *2*(12), e214854.

4 Emanuel et al., op. cit. note 1.

5 Borio, L. L., Bright, R. A., & Emanuel, E. J. (2022). A national strategy for COVID‐19 counter‐measures: Vaccines and therapeutics. *Journal of the American Medical Association*, *327*, 215–216. Epub ahead of print, January 6: https://doi.org/10.1001/jama.2021.24165

6 Johns Hopkins University, Center for Communication (2021). *COVID behaviors dashboard*. Retreived December 22 from: https://covidbehaviors.org/#who-is-most-willing-to-accept-a-vaccine-within-a-particular-country

7 U.S. Centers for Disease Control and Prevention. (2021). COVID‐19 vaccine breakthrough case investigation team, 2021. COVID‐19 vaccine breakthrough infections reported to CDC ‐ United States, January 1‐April 30. *Morbidity and Mortality Weekly Report*, *70*(21), 792–793.

8 Andrews, N., Stowe, J., Kirsebom, F., Toffa, S., Rickeard, T., Gallagher, E., … Bernal, J. L. (in press). *Effectiveness of COVID‐19 vaccines against the Omicron (B.1.1.529) variant of concern*. 135939561/43098, 2021.6542/Effectiveness+of+COVID‐19+vaccines+against+Omicron+variant+of+concern.pdf/f423c9f4‐91cb‐0274‐c8c5‐70e8fad50074

9 Adamson, B., Sikka, R., Wyllie, A. L., & Premsrirut, P. (2022). Discordant SARS‐CoV‐2 PCR and rapid antigen test results when infectious: A December 2021 occupational case series. *medRxiv*, preprint, January 5: https://doi.org/10.1101/2022.01.04.22268770

10 Jecker, N. S., & Atuire, C. A. (2021). What's yours is ours. *Journal of Medical Ethics*, *47*, 595–598. https://doi.org/10.1136/medethics-2021-107555

11 Planas, D., Saunders, N., Maes, P., Guivel‐Benhassine, F., Planchais, C., Buchrieser, J., … Schwartz, O. (2022). Considerable escape of SARS‐CoV‐2 variant omicron to antibody neutralization. *Nature 602*, 671–675. https://doi.org/10.1038/s41586-021-04389-z; Gupta, P. C., Aggarwal, S., Jain, P., Jugran, D., Sharma, M., Pandav, S. S., & Ram, J. (2021). SARS‐CoV‐2 omicron: Evasion of potent humoral responses and resistance to clinical immunotherapeutics relative to viral variants of concern. *medRxiv. Preprint*, 15 December, *69*, 3648–3650. https://doi.org/10.1101/2021.12.14.21267772

12 GlaxoSmithKline. (2021, 2 Dec). *Preclinical data demonstrate sotrovimab retains activity against key omicron mutations, new SARS‐CoV‐2 variant*. Retrieved from: https://www.gsk.com/en-gb/media/press-releases/sotrovimab-retains-activity/

13 Moodley, K., Rennie, S., & Behets, F. (2020). Allocation of scarce resources in Africa during COVID‐19. *Developing World Bioethics*, *21*, 36–43. ePub ahead of print: https://doi.org/10.1111/dewb.12280; Stein, F., Perry, M., Banda, G., Woolhouse, M., & Mutapi, F. (2020). Oxygen provision to fight COVID‐19 in sub‐Saharan Africa. *BMJ Global Health*, *5*(6), e002786.

14 Emanuel, E. J., Persad, G., Kern, A., Buchanan, A., Fabre, C., Halliday, D., … Richardson, H. S. (2020). An ethical framework for global vaccine allocation. *Science*, *369*(6509), 1309–1312; Jecker, N. S., Wightman, A. G., & Diekema, D. S. (2021). Vaccine ethics: An ethical framework for global distribution of COVID‐19 vaccines. *Journal of Medical Ethics*, *47*, 308–317; Jecker, N. S., & Atuire, C. A. (2021). Out of Africa: A solidarity‐based approach to vaccine allocation. *Hastings Center Report*, *51*(2), 27–36; World Health Organization. (2020). *WHO/SAGE values framework for the allocation and prioritization of COVID‐19 vaccination*. Retrieved from: https://apps.who.int/iris/bitstream/handle/10665/334299/WHO-2019-nCoV-SAGE_Framework-Allocation_and_prioritization-2020.1-eng.pdf?sequence=1%26isAllowed=y; World Health Organization. (2020). *Fair allocation mechanism for COVID‐19 vaccines through the COVAX facility*. Retrieved from: https://www.who.int/publications/m/item/fair-allocation-mechanism-for-covid-19-vaccines-through-the-covax-facility

15 Cohen, P., & Blau, J. (2021, Dec 6). COVID‐19: Outpatient evaluation and management of acute illness in adults. *UpToDate*. Retreived from: www.uptodate.com

16 Francis, L. P., Battin, M. O., Jacobson, J. A., Smith, C. B., & Botkin, J. (2005). How infectious disease got left out and what this omission might have meant for bioethics. *Bioethics*, *19*(307–322), 313–322.

17 Oxford University Press. (2021). “solidarity, n.” *In OED Online*. Oxford University Press.

18 West‐Oram, P. G. N., & Buyx, A. (2017). Global health solidarity. *Public Health Ethics*, *10*(2), 212–224.

19 Prainsack, B., & Buyx, A. (2017). Solidarity in biomedicine and beyond(p. 52). Cambridge University Press.

20 West‐Oram & Buyx, op. cit. note 18, p. 213.

21 Hussain, W. (2018). Common good. In E. N. Zalta (Ed.), *Stanford encyclopedia of philosophy*. Stanford University Press. Retrieved from: https://plato.stanford.edu/archives/spr2018/entries/common-good/

22 Parfit, D. (1997). Equality and priority. *Ratio*, *10*, 202–221.

23 Bell, B. P., Romero, J. R., & Lee, G. M. (2020). Scientific and ethical principles underlying recommendations from the Advisory Committee on immunization practices for COVID‐19 vaccination implementation. *Journal of the American Medical Association*, *324*(20), 2025–2026; National Academies of Science, Engineering and Medicine. (2020). *framework for equitable allocation of COVD‐19 vaccines*. National Academies Press.

24 World Health Organization, op. cit. note 14; Jecker et al., op. cit. note 14.

25 Goldin, I., & Mariathasan, M. (2015). *The butterfly defect* (p. 1). Princeton University Press.

26 Jecker & Atuire, op. cit. note 14.

27 Callaway, E. (2021). Delta coronavirus variant. *Nature*, *595*, 17–18.

28 Jecker, N. S. (2020). African conceptions of age‐based moral standing. *Hastings Center Report*, *50*(2), 35–43.

29 Schaefer, G. O., Leland, R. J., & Emanuel, E. J. (2021). Making vaccines available to other countries before offering domestic booster vaccinations. *Journal of the American Medical Association*, *326*(10), 903–904.

30 Jecker, N. S., & Lederman, Z. (2021). Three for me and none for you? An ethical argument for delaying COVID‐19 boosters. Journal of Medical Ethics. Epub ahead of print: https://doi.org/10.1136/medethics-2021-107824

31 Schaubroeck, K., & Hens, K. (2021). Pandemic risk and standpoint epistemology. Health Care Analysis. Epub ahead of print: https://doi.org/10.1007/s10728-021-00443-z

32 Yankelovich, D. (1991). Coming to public judgment. Syracuse University Press.

33 Jecker, N. S., & Atuire, C. A. (2021, 30 November). Who's in? Who's out? The ethics of COVID‐19 travel rules. *The Conversation*. Retrieved from: https://theconversation.com/whos-in-whos-out-the-ethics-of-covid-19-travel-rules-172053

34 Chinazzi, M., Davis, J. T., Ajelli, M., Gioannini, C., Litvinova, M., Merler, S., Pastore y Piontti, A., Mu, K., Rossi, L., Sun, K., Viboud, C., Xiong, X., Yu, H., Halloran, M. E., Longini, I. M., & Vespignani, A. (2020). The effect of travel restrictions on the spread of the 2019 novel coronavirus (COVID‐19) outbreak. *Science*, *368*, 395–400.

35 Moeti, M. (2021, 28 November). *Quoted in WHO, WHO stands with African nations and calls for borders to remain open*. Retrieved from: https://www.afro.who.int/news/who-stands-african-nations-and-calls-borders-remain-open

36 Ghebreyesus, T. A. (2021, Dec 22). *Quoted in World Health Organization, WHO warns against blanket boosters as vaccine inequity persists*. Retrieved from: https://news.un.org/en/story/2021/12/1108622.

37 Jecker & Atuire, op. cit. note 30.

38 Persad, G., Parker, W. F., & Emanuel, E. J. (2021, Apr 8). Taming the 4th COVID surge. *USA Today*.

39 Hassoun, N. (2021). Good enough? The minimally good life account of the basic minimum. Australasian Journal of Philosophy. EPub ahead of print: https://doi.org/10.1080/00048402.2021.1905674; Hassoun, N. (2021). Sufficiency and the minimally good life. *Utilitas*, 33, 321–336. https://doi.org/10.1017/S0953820820000497

40 Gunson, D. (2009). Solidarity and the universal declaration on bioethics and human rights. *Journal of Medicine and Philosophy*, *34*, 241–260, 247.

41 Nolen, S. (2021, Nov 4). Merck will share formula for its covid pill with poor countries. *New York Times*.

42 Scheffler, S. (2002). Boundaries and allegiances. Oxford University Press.

43 National Academies of Science, Engineering and Medicine, op. cit. note 23, p. 20.

44 Gould, C. (2018). Solidarity and the problem of structural injustice. *Bioethics*, *32*, 541–552, 545.

45 Jecker, N. S. (2022). Global sharing of COVID‐19 vaccines: A duty of justice, not charity. Developing World Bioethics. Epub ahead of print: https://doi.org/10.1111/dewb.12342

46 Makridis, C. A., Wu, C., & Freeman, J. (2021). How social capital helps communities weather the COVID‐19 PANDEMIC. *PLoS One*, *16*(1), e0245135. https://doi.org/10.1371/journal.pone.0245135

47 Miller, D. (2021). Justice. In E. N. Zalta (Ed.), *Stanford encyclopedia of philosophy*. Stanford University Press. Retrieved from: https://plato.stanford.edu/archives/fall2021/entries/justice/

48 West‐Oram & Buyx, op. cit. note 18.

49 Gostin, L. O., Monahan, J. T., Kaldor, J., DeBartolo, M., Friedman, E. A., Gottschalk, K., … & Yamin, A. E. (2019). The legal determinants of health: Harnessing the power of law for global health and sustainable development. *Lancet*, *393*(1857–1910), 1858–1910.

50 European Council. (2020, Dec 3). *Press release by President Charles Michel on an International Treaty on pandemics*. Retrieved from: https://www.consilium.europa.eu/en/press/press-releases/2020/12/03/press-release-by-president-charles-michel-on-an-international-treaty-on-pandemics/

51 Ten Have, H. (2016). Global bioethics (p. 9). Routledge.

52 Jecker, N. S., Wightman, A. G., & Diekema, D. S. (2020). Prioritizing frontline workers during the COVID‐19 pandemic. *American Journal of Bioethics*, *20*(7), 128–132.

53 Kleingeld, P., & Brown, E. (2019). Cosmopolitanism. In. E. N. Zalta (Ed.), *Stanford encyclopedia of philosophy*. Stanford University Press. Retrieved from: https://plato.stanford.edu/archives/win2019/entries/cosmopolitanism/

54 Corey, L., Beyrer, C., Cohen, M. S., Michael, N. L., Bedford, T., & Rolland, M. (2021). SARS‐CoV‐2 variants in patients with immunosuppression. *New England Journal of Medicine*, *385*, 562–566.

55 Emanuel, E. J., Buchanan, A., Chan, S. Y., Fabre, C., Halliday, D., Heath, J., … & Persad, G. (2021). What are the obligations of pharmaceutical companies in a global health. *Lancet*, *398*, 1015–1020. Epub ahead of print: https://doi.org/10.1016/S0140-6736(21)01378-7

